# A CEO’s Future Temporal Depth and Organizational Resilience

**DOI:** 10.1007/s41471-022-00145-9

**Published:** 2022-11-18

**Authors:** Maximilian Weis, Patricia Klarner

**Affiliations:** Vienna University of Economics and Business, Welthandelsplatz 1, 1020 Vienna, Austria

**Keywords:** CEO temporal cognition, Upper Echelons, Organizational crisis, Organizational resilience

## Abstract

Scholars have long investigated the organizational antecedents of resilience, but less is known about CEO-level antecedents. This is surprising, since upper echelons research suggests that a CEO influences major firm decisions. Addressing this gap in our knowledge, we suggest that a CEO prepares for and adjusts to unexpected events in the environment on the basis of the individual future temporal depth (FTD). It reflects the temporal distance into the future that a CEO usually takes into consideration when contemplating future events. Our study of CEOs of 462 S&P500 firms during the Global Financial Crisis and the COVID-19 pandemic shows that a CEO’s longer FTD is associated with less severe economic losses but with a longer recovery time from adversity. If such a CEO can draw on a functionally diverse TMT, the losses are less severe, while prior organizational crisis experience reduces the recovery time. Our paper contributes to organizational resilience research by uncovering its cognitive underpinnings and offering a contextual learning perspective on organizational resilience. We also contribute to upper echelons research by unveiling a CEO’s role in preparing for and adjusting to adversity.

## Introduction

Research on organizational resilience has long suggested that developing resilience is a long-term organizational undertaking (Ortiz-de-Mandojana and Bansal 2016; Williams et al. 2017; DesJardine et al. 2019). It stems from a firm’s ability to persist despite disruptions (i.e., its stability), and to regenerate (i.e., its flexibility) (DesJardine et al. 2019; Aven 2011). First, organizational resilience allows firms to endure major disruption by processing external signals and responding to them (Vogus and Sutcliffe 2003), and, second, to bounce back by applying a wide range of crisis responses (Sanchez 1995). While developing such routines and practices (Reinmoeller and Van Baardwijk 2005), building up slack resources (Gittell et al. 2006), and establishing governance mechanisms (Carmeli and Markman 2011) take time, fast and decisive reactions and responses are critical in the face of adversity.

Prior research has mainly focused on organizational-level antecedents of organizational resilience, such as flexible financial resources (Gittell et al. 2006) or sustainable business practices (Ortiz-de-Mandojana and Bansal 2016), far fewer studies have examined a CEO’s role (Buyl et al. 2019; Sajko et al. 2021). This is surprising because upper echelons theory has long suggested that top executives’—and particularly the CEO’s—characteristics and experiences influence strategic actions and firm outcomes (Hambrick 2007; Buyl et al. 2011b). Extant research, for instance, shows that CEO narcissism (Buyl et al. 2019) and CEO greed (Sajko et al. 2021) affect a firm’s recovery from a crisis. These first insights confirm that managers’ personality traits and individual desires influence executive decision making during crises. While extant research emphasizes the distinct mechanisms that enable organizational resilience (i.e. the long-term preparation for and the short-term response to adversity), it has not yet uncovered temporality’s role in organizational resilience and how it relates to different time horizons. Consequently, we surmise that how a CEO prepares for and adjusts to adversity should reflect a CEO’s time horizons. Indeed, there is evidence that individuals’ strategies for dealing with crises or change are based on the time frames they adopt (Mumford et al. 2007). These temporal considerations are reflected in a CEO’s future temporal depth (FTD), which refers to the temporal distance into the future that a CEO usually takes into consideration when contemplating future events (Bluedorn 2002; Nadkarni et al. 2016). In other words, the FTD determines how short- or long-term oriented a CEO is. Scholars have started examining a CEO’s future temporal depth in different strategic choices, such as strategic decision making (Das and Teng 2001; Lin et al. 2019) and competitive aggressiveness (Nadkarni et al. 2016), but have paid little attention to how it influences the preparation for and dealing with crises.

In this paper, we integrate research on organizational resilience (Hillmann and Guenther 2021; Conz and Magnani 2020) and upper echelons (Hambrick 2007; Buyl et al. 2019). Doing so we examine the role that a CEO’s FTD plays in organizational resilience, unveiling its managerial cognitive underpinnings. The organizational duality of stability (i.e. to withstand adversity and survive) and flexibility (i.e. to respond to crises and adapt to new situations) reflects organizational resilience, which we operationalize as the severity of the loss and the time the relevant stock price requires to recover (DesJardine et al. 2019). The key notion is that stock prices react to novel information about a company and its activities. The *severity of the loss* refers to the extent of a stock performance’s decline after a crisis. The severity of a loss relates to a crisis’s immediate impact, uncovers a firm’s vulnerability to unexpected disturbances, and reflects its ability to maintain stability in the face of adversity (Weick et al. 2008). *Time to recovery *refers to the time a firm requires to recover to the pre-shock stock price levels and relates to its flexibility (Ortiz-de-Mandojana and Bansal 2016; DesJardine et al. 2019). We propose that longer CEO FTD is associated with less severe economic losses, but slower recovery from an external shock than a shorter CEO FTD.

We offer a contextual learning perspective on organizational resilience by introducing a TMT’s functional experience diversity and organizational crisis experience as vital contingencies of how a CEO prepares for and adjusts to adversity. While a CEO contributes to organizational resilience by processing and reacting to external signals (Vogus and Sutcliffe 2003), the CEO also depends on the TMT to make effective decisions in the face of adversity (Finkelstein et al. 2009; Hambrick 2007). The TMT is the team of top executives who act as an interface between the firm and its environment (Van Doorn et al. 2022). They “are relatively powerful, and therefore their choices and actions are likely to have an impact on the organization” (Carpenter et al. 2004: 753). The TMT’s diversity of functional experiences allows a CEO to better collect and evaluate multiple information sources across departments, which eventually improves the CEO’s decision making (Mohammed and Harrison 2013). We therefore suggest that a CEO with a longer FTD is more likely to draw on the TMT’s functional diversity, the multitude of views and different function-specific solutions before crises, such that a functionally diverse TMT’s presence is associated with a decrease in the severity of loss.

Prior research suggested that an organization can develop resilience by learning through experience with adversity (Williams et al. 2017; Weick et al. 2008). A better understanding of what causes crises and opportunities to learn from past crisis events allow firms to respond to them more effectively (Kovoor-Misra and Nathan 2000). We suggest that a CEO with a longer FTD is more likely to benefit from prior organizational crisis experiences, therefore decreasing the time to recovery. Organizational crisis experience fosters organizational flexibility by providing a set of prior responses and timely reactions to adversity, which enables a CEO with a longer FTD to respond more rapidly.

By applying a novel machine learning (ML) approach to measure a CEO’s FTD in conference calls with analysts, we tested our predictions during the 2008 Global Financial Crisis (GFC) and the 2020 COVID-19 pandemic with a sample of CEOs of 462 S&P500 firms. Our findings confirmed that a longer CEO FTD is associated with less severe economic losses, but a longer recovery time. However, if such a CEO can draw on a highly diverse TMT the losses are less severe, while the recovery is faster if the firm has organizational crisis experience.

Our study offers a more fine-grained understanding of organizational resilience and how a CEO prepares for and adjusts the organization when it faces adversity. We contribute to organizational resilience research (Ortiz-de-Mandojana and Bansal 2016; DesJardine et al. 2019) by uncovering its cognitive underpinnings, thereby unveiling the important role that a CEO’s FTD plays. We offer a contextual learning perspective on organizational resilience by emphasizing TMT experience diversity’s and organizational crisis experience’s different effects. We also contribute to upper echelons research (Nadkarni et al. 2016; Chen and Nadkarni 2017) by emphasizing the important role that a CEO’s FTD plays in organizational resilience, and the importance of a functionally diverse TMT working with a CEO to steer an organization through crises. Lastly, we apply state-of-the-art ML techniques (Menon et al. 2018) to develop a novel measure of a CEO’s FTD, thereby laying the foundation for future research on a CEO’s temporal cognition influencing strategic and organizational decisions.

## Literature

### Organizational Resilience

The COVID-19 pandemic and the GFC are more recent examples of crises defined as threatening and stressful external events causing organizations financial and economic distress (DesJardine et al. 2019). Such crises emerge from impactful, quick-developing events, such as natural disasters, pandemics, and economic downturns (Williams et al. 2017; Zhou et al. 2010). Organizational resilience, which is a firm’s ability to persist and regenerate in the face of adversity (DesJardine et al. 2019; Aven 2011), is crucial in these contexts. Resilience allows a firm to, first, endure major disruption by processing and responding to external signals (Vogus and Sutcliffe 2003), and, second, to bounce back by applying a wide range of alternative responses (Sanchez 1995). Since organizational resilience is a latent construct, which is difficult to observe directly, scholars tend to estimate it by means of its effect on financial and firm outcomes, such as the stock price’s recovery from external adversity (Gittell et al. 2006), or the growth in sales (Ortiz-de-Mandojana and Bansal 2016).

To explain the variation in firms’ abilities to respond to and endure adversity, organizational resilience research often emphasizes relevant pre-crisis attributes (Van Der Vegt et al. 2015), such as slack resources or the willingness to question what is happening (Vogus and Sutcliffe 2003). Extant research suggests that flexible financial resources or slack (Gittell et al. 2006), innovation practices (Reinmoeller and Van Baardwijk 2005), and good governance (Carmeli and Markman 2011) could have a positive impact on organizational resilience. Gittell et al. (2006), for example, studied the United States’ airline industry after the 9/11 terror attacks, finding that the airlines that had amassed the most financial reserves and avoided high levels of debt recovered more quickly, even exceeding their earlier performance levels without laying-off employees. Moreover, by studying 195 firms on the KLD 400 index from 1994 to 2008, Ortiz-de-Madojana and Bansal (2016) found that social and environmental practices contribute to lower volatility, higher sales growth, and survival. While most research has studied several employee-level (e.g., Shin et al. 2012; Youssef and Luthans 2007) or organizational-level (e.g., DesJardine et al. 2019) antecedents of organizational resilience, far fewer studies have examined a CEO’s role in it.

### A CEO’s Role in Organizational Resilience

Upper echelons theory suggests that how top executives process information shapes their strategic choices (Hambrick 2007). This perspective emphasizes three stages in the information filtering process of top executives (Steinbach et al. 2019; Finkelstein et al. 2009): (1) top executives only possess a limited vision within which they direct their attention (Hambrick et al. 1996), (2) they have a selective perception of what stimuli to consider further (Waller et al. 1995), (3) and they interpret information through a filter by their cognitive bases and values (Hambrick and Mason 1984). The result of this filtering process is that no two CEOs interpret the same stimuli in an identical manner, which leads to different strategic decisions despite being confronted by similar situations (Finkelstein et al. 2009). Especially in times of crises, when CEOs tend to simplify complex tasks and deal with uncertainty, these mechanisms shape how CEOs interpret and react to situations (Hodgkinson and Healey 2008; Dutton 1986).

First scholars have started to uncover these mechanisms in organizational resilience from a CEO personality perspective. For instance, in a study of 92 U.S. commercial banks, Buyl et al. (2019) found that CEO narcissism harms resilience, such that a firm headed by a more narcissistic CEO before the GFC faced a slower recovery to pre-crisis performance levels. Similarly, Sajko et al. (2021) studied 269 S&P1500 CEOs, finding that CEO greed and CSR did not affect the drop in a firm’s shares, but that it did take longer to recover. These first insights confirm that a CEO’s personality and desires are determinants of executive decision making before and during crises.

However, it is surprising that little research has yet studied how CEO cognition, particularly a CEO’s temporal cognition, affect organizational resilience. Especially since prior research stresses the distinct mechanisms that enable organizational resilience (DesJardine et al. 2019): While building stability for adversity takes time and requires continuous preparation, flexibility requires responding and reacting quickly to changes in the organization and the environment. These two dimensions both have distinct temporal aspects. Therefore, we suggest that a CEO’s temporal cognition plays an important role in filtering and interpreting information related to the time-sensitive aspects of resilience, thus influencing important decisions.

Indeed, prior research has shown that a CEO’s temporal cognition affects the CEO’s thinking and behavior fundamentally (Lin et al. 2019). Research on the subjective perception of time in particular proposes that an individual’s interpretation of time operates as a cognitive lens that shapes the understanding and evaluation of situations and depicts the basis for strategic actions (Ancona et al. 2001; Chen and Nadkarni 2017; Shipp and Cole 2015). Ignoring these individual temporal attributes would lead to “a misleading conception of time when it relates to strategy making” (Das 2004: 59). Dating back to Lewin’s (1942) view of time, extant research suggests that the subjective perspective of time has important implications of how individuals make decisions (Shipp and Cole 2015; Nadkarni and Chen 2014). Two temporal constructs have gained increasing momentum: temporal focus and temporal depth. *Temporal focus* reflects an individual disposition to which individuals “characteristically direct their attention to the past, present, or future” (Shipp and Cole 2015: 244). How CEOs think about time is critical in understanding their strategic decisions and actions (Das 2004; Nadkarni and Chen 2014). Extant research, for instance, studied the role of CEO’s temporal focus in resource deployment (Yadav et al. 2007), new product introduction (Nadkarni and Chen 2014), and media reactions (Gamache and McNamara 2019). While a CEO’s temporal focus refers to the emphasis on the past, present, or future (Bluedorn 2002), a CEO’s *temporal depth* reflects the temporal distance into the past and the future a CEO considers (Nadkarni et al. 2016).

In this study, we focus on a CEO’s future temporal depth (FTD), which refers to the temporal distance that CEOs consider when thinking about future events (Bluedorn 2002; Nadkarni et al. 2016). According to social psychology research, temporal depth is *relatively* stable over time (Marin 1987)*.* In other words, a FTD describes whether a CEO is more short-term (e.g., focusing on quick returns, near-term actions, and outcomes) or long-term oriented (e.g., focusing on more distal outcomes, having a future vision). From an upper echelons perspective (Hambrick and Mason 1984; Hambrick 2007) this has severe implications on the information filtering of these CEOs, the time horizons they consider relevant, and which actions they pay attention to. For instance, CEOs with a high FTD have a clearer vision and longer range set of plans in place (Das 1987), paying attention to future opportunities and signals.

Extant research on individual future temporal depth mainly focuses on strategic outcomes, such as companies’ competitive aggressiveness (Nadkarni et al. 2016), and strategic decision making (Das and Teng 2001). While a longer FTD fosters comprehensiveness and creativity in strategic decision making (Lin et al. 2019), a shorter FTD relates to flexible and spontaneous decision making (Bluedorn 2002) and an emphasis on the “here and now” (Nevins et al. 2007). A CEO’s FTD determines an organization’s strategic planning (Das 1987), but also the pace of this decision making (Lin et al. 2018). These are crucial aspects of preparing for, but also rapidly adjusting to external adversity. We, therefore, study the role of a CEO’s FTD in both dimensions of organizational resilience: stability and flexibility.

## Theory and Hypotheses

### A CEO’s Future Temporal Depth and Stability

A firm’s ability to persist despite disruptions defines one dimension of organizational resilience. We address this by studying the *severity of a loss,* which is the maximum economic loss that a firm faces soon after an external shock or adversity, therefore reflecting a firm’s *stability*. A firm facing less severe losses mirrors a more stable system since it is better at absorbing a shock (Weick et al. 2008). By maintaining their ability to operate, such firms anchor their activities, protecting themselves from environmental and external disturbances’ harm, thereby avoiding new unfavorable states, such as potential bankruptcy (DesJardine et al. 2019). This requires constant preparation before the actual crisis hits (Williams et al. 2017). We surmise that a CEO’s longer FTD is associated with less severe losses for the following reasons:

First, to prepare the organization for potential adversity, the CEO needs to detect potential warning signs and interpret them accurately to mobilize organizational attention and resources (Chong 2004; Maitlis and Sonenshein 2010). How a CEO filters this information and makes decisions is influenced by the CEO’s cognitive processes (Finkelstein et al. 2009; Hambrick and Mason 1984). A CEO with a longer FTD is able to process large amounts of information (Bluedorn 2002; Nadkarni et al. 2016), since a longer FTD is associated with better pattern recognition abilities (Bluedorn and Martin 2008). This enables a CEO to scan the organization and the environment, recognize and interpret potential problems and threats (Marcus and Nichols 1999). Detecting signs early that might escalate into disaster if a firm is not prepared (Weick 2003), and a comprehensive interpretation of information helps building stability in the organization (Ortiz-de-Mandojana and Bansal 2016).

Second, a CEO with a longer FTD takes a wider range of stakeholders into account (Wang and Bansal 2012). Engagement with stakeholders builds stability by developing interlinkages and reinforcing the relationship between different stakeholders and the firm (Simpson et al. 2013). Since a CEO with a longer FTD specifically invests in building long-lasting, stable relationships with others (Joireman et al. 2006), such a CEO likely benefits from recurrent exchanges with relevant stakeholders and from frequently sharing information and knowledge (Albert et al. 2015). Stakeholders are willing to share sensitive information about emerging issues with a trusted CEO if they believe the CEO will respond to the comments and not use the information against them (Harrison et al. 2010). This finding is due to a longer FTD being associated with prosocial behaviors (Zimbardo and Boyd 1999) and more inclusive social networks (Carstensen et al. 1999). Having access to a wide range of stakeholders helps such a CEO to effectively notice “weak signs of danger” (e.g., a change in performance), and to push these signals onto the TMT’s strategic agenda, emphasizing the need to organize differently, and supporting open discussions about proactive measures (Bavik et al. 2021).

Third, to build up stability, reduce the severity of external shocks, a CEO needs to constantly prepare the organization by improving its capacity for resilience and making it resistant to economic disruption (de Oliveira Teixeira and Werther 2013). Such preparation for adversity is reinforced by long-term strategic thinking. A CEO with a longer FTD prepares the firm for external disturbances by deciding on priorities and objectives that will show results over an extended period and by paying special attention to these (Le Breton-Miller and Miller 2006). Such a CEO, for example, tends to implement longer-term strategic planning horizons (Das 1987, 1991). With a vision of the organization, a CEO may enact a future narrative that contextualizes threats and develops future-oriented stories. Such a CEO promotes an “ontology of possibility,” which builds resilience (Lawrence and Maitlis 2012: 641).

Since a CEO with a longer FTD scans information more broadly, builds and develops trustful relationships with several stakeholders, and constantly prepares for the future, we suggest that a longer FTD builds stability. Stability is reflected in a constant preparation of the organizations for potential adversity, therefore likely resulting in less severe losses in times of crisis. We therefore propose.

#### Hypothesis 1

A CEO’s longer FTD is negatively associated the severity of an organization’s loss due to an external shock.

### A CEO’s Future Temporal Depth and Flexibility

A firm’s ability to regenerate is another dimension of resilience. We address this by studying the *time to recovery*, which is the amount of time that a firm requires to return to its original, pre-crisis state, reflecting the firm’s *flexibility*. A firm with a shorter recovery time suggests a more flexible system, because it is better able to adjust to adversity. Flexibility allows firms to adapt faster to external disruptions (Brand and Jax 2007) and to absorb disturbances “while transforming their structures and means for functioning in the face of long-term stresses, change, and uncertainty” (Van Der Vegt et al. 2015: 972). We propose that a CEO’s longer FTD increases the firm’s time to recovery for the following reasons:

First, fast-developing crises often develop across all economic levels and tend to be very disruptive (Bonanno et al. 2010). When facing high levels of adversity, a CEO needs to make time-sensitive choices between “staying the course” and departing from intended routes. Despite the crucial role that planning and preparing for external adversity play, extensive planning and preparation cannot lessen all external shocks (Neal and Phillips 1995; Drabek and McEntire 2003; Herbane 2013). In a crisis, a CEO has to make decisions, gather information and process it to take actions that are aligned with the firm’s strategic priorities promptly (Chong 2004; Pearson and Clair 1998). Therefore, a CEO whose cognitive responses help direct attention correctly, allows for concentrating on the most suitable available alternatives to decrease complexity, and develop novel, alternative future actions (Lengnick-Hall and Beck 2005). A CEO with a longer FTD is more likely to emphasize long-term actions rather than more near-term ones. In other words, a CEO’s long FTD may lead to being hesitant to deviate from a long-term vision, which can create rigidities that can hinder a CEO to recognize changes in the short-term environmental conditions and create inertia in adapting to them (Levinthal and March 1993). Therefore, a CEO with a longer FTD likely takes more time to adapt to such changes, resulting in slower firm recovery.

Second, when exposed to major disturbances, a CEO needs to improve the firm’s condition by redistributing its assets, altering its internal controls, and improving the coordination mechanisms between businesses (Barbero et al. 2017; Tangpong et al. 2015). In such situations, a CEO faces amplified uncertainty about how to react (Duncan 1972; Milliken 1987) and needs to generate a variety of potential answers fast. In line with this action, prior research highlighted the crucial role that flexible resources, stored in anticipation of unexpected crises and adversity, can play when applied at such a time (Carmeli and Markman 2011; Virany et al. 1992; George 2005). Adequate internal resources, as well as the ability to rearrange and adjust these resources, are vital to allow a firm to recover its performance level after external adversity (Bayazitova and Shivdasani 2012; Vogus and Sutcliffe 2003). However, a CEO with a longer FTD favors future benefits over quick results and prompt solutions (Das 1987), which may lead such a CEO to selectively focus attention on future issues neglecting near team signals. As a result, a CEO with a longer FTD may take more time to realize and acknowledge the need for actual and prompt action—despite having prepared the organization for crisis.

Since a CEO with a longer FTD focuses on future outcomes instead of present ones and hesitates to deviate from set plans, this may limit a firm’s ability to recover fast in the face of an unexpected external shock. We therefore propose,

#### Hypothesis 2

A CEO’s longer FTD increases the time an organization needs to recover due to an external shock.

In the following, we offer a contextual learning perspective on organizational resilience by introducing TMT functional experience diversity and organizational crisis experience as vital contingencies of how a CEO prepares for and adjusts to adversity. While a CEO contributes to organizational resilience by processing and reacting to external signals (Vogus and Sutcliffe 2003), a CEO also depends on the TMT (Finkelstein et al. 2009; Hambrick 2007) and prior organizational crises experience. We surmise that a TMT’s functional diversity contributes to an organization’s stability by allowing the members to process and share multiple experiences and viewpoints. Organizational crisis experience fosters organizational flexibility by providing a pool of prior responses and timely reactions to adversity.

### Moderating Effect of TMTs’ Functional Experience Diversity

A CEO’s longer FTD likely decreases the severity of loss by allowing the CEO to process large amounts of information (Bluedorn 2002; Nadkarni et al. 2016) and to decide on priorities and objectives that will show results over an extended period (Le Breton-Miller and Miller 2006). Given the complexity that many firms face, a CEO alone might not be able to successfully scan the external environment for information. In particular, TMT members with diverse experience and skills often contribute significantly to strategic decision-making (Nielsen and Nielsen 2011). The diversity of the TMT members’ functional experience is a crucial source of top executives’ know-how (Buyl et al. 2011a; Bunderson and Sutcliffe 2002). TMT functional diversity determines the stimuli to which a TMT is sensitive, the opportunities it perceives and takes, and the interpretations each member brings to task-related discussions (Cho and Hambrick 2006). TMT members’ experiences can be vital when dealing with complex situations such as crises (Edmondson et al. 2003), and when the entire TMT needs to engage in scanning efforts (Cho 2006). We expect that a functionally diverse TMT’s rich pool of experiences further prepares for crisis. Particularly, we presume that a TMT’s functional experience diversity strengthens the relationship between a CEO’s longer FTD and the severity of loss, such that a functionally diverse TMT’s presence is associated with less severe losses.

Diverse teams are more likely to perceive shifts in their external environment (Sutcliffe and Vogus and Sutcliffe 2003). Cho (2006), for instance, showed that TMTs with a high functional diversity scan complex environments better. Such a broader scanning could help a CEO with a longer FTD who focuses on the more temporally distal requirements and signals to scan and prepare for potential threats and provide a broader range of relevant function-specific information. Moreover, higher levels of functional diversity lead to increased TMT cognitive diversity, which could improve the team’s predictive and forecasting ability—an essential ability in complex environments (Page 2014). Functionally diverse teams cannot reduce the complexity or the amount of unexpected events. Nevertheless, the team members’ diversity could produce a more diverse pool of ideas, which “should lead to less collective surprise and therefore a better preparation for the consequences” (Page 2014: 275), which allows for broader information screening and processing. TMT diversity improves problem solving by extending its abilities to search for alternative answers (Eisenhardt and Schoonhoven 1990). By reconciling and including diverse perspectives, this breadth of functional experience could be especially helpful when dealing with complex and rare challenges (Van Knippenberg and Schippers 2007).

Since a CEO is the guardian of decision processes for the TMT, a CEO can encourage or dampen open debate (Cannella and Holcomb 2005). Longer FTD is associated with more prosocial behavior and openness to others’ views (Wojtkowska et al. 2021). A CEO with a long FTD is thus more likely to be open to and incorporate the TMT’s functional experience diversity. Consequently, TMT functional diversity helps a CEO with a longer FTD by providing a more fine-grained scanning of the cross-functional range of possible risks and developing possible solutions to address them (Duchek et al. 2020). We propose,

#### Hypothesis 3

A CEO with a longer FTD who can draw on a functionally diverse TMT is more negatively associated with the severity of loss due to an external shock.

### Moderating Effect of Organizational Crisis Experience

A CEO’s longer FTD likely increases the time to recovery due to less flexible decision making and a hesitance to deviate from set plans. Yet, to recover to pre-crisis stock price levels, the organization’s accumulated experience in dealing with adverse signals and deriving corresponding approaches to respond to them is important (Madsen and Desai 2010)—in addition to a CEO’s longer FTD. We argue that organizational crisis experience provides the cumulative knowledge and skills that can support a CEO with a longer FTD to recover faster in the face of adversity. We expect that prior organizational crisis experience mitigates the effect of a CEO’s longer FTD on the time to recovery, such that the recovery time decreases in the presence of prior organizational crisis experience.

Crisis experience builds awareness of weaknesses within the organization and can lead to a quicker assessment and addressing of these issues. For instance, Madsen and Desai (2010) suggest that crisis experience fosters a probabilistic search for the causes of and solutions for adversity. Organizations become collectively sensitive to organizational blind spots through the experiences and routines acquired during prior crises. This also shapes the form of cognitive reasoning in top executives’ and other organization members’ mental models (Deverell and Olsson 2009), which is particularly useful for a CEO with a longer FTD. Such a CEO requires a deeper understanding of the organization and often tends to connect with organizational members and engage in open networks (Opper and Burt 2021). This makes it easier for the CEO to tap into that knowledge and experience gained from prior crises. Since crises require a firm to rapidly find adequate solutions for often novel and unexpected problems (Vogus and Sutcliffe 2003), they can trigger a focus on generating and collecting novel skills and knowledge (Christianson et al. 2009). Zahra and George (2002), for instance, proposed that organizational crisis experience could increase employees’ learning abilities. A CEO with a longer FTD can thus benefit from the organization’s past crisis responses, assessing which responses have been feasible and where potential resistance among employees may reside. Prior crisis experience may also help such CEOs garner more support for effective crisis response, potentially even pushing a CEO with a longer FTD towards faster responses than such a CEO envisaged. All of these aspects allow a CEO with a longer FTD to draw on a set of accumulated experiences in dealing with crises, eventually decreasing the recovery time. We propose,

#### Hypothesis 4

A CEO with a longer FTD who can draw on organizational crisis experience decreases the organization’s time to recover due to an external shock.

## Sample

### Setting and Timing

We study organizational resilience during two crises: the GFC and the COVID-19 pandemic. The GFC was one of the worst crises since the Great Depression (Brunnermeier 2009), with global equity markets falling by 59% ($37 trillion) in roughly 18 months (Anand et al. 2013). Despite the GFC’s triggering events originating in the U.S., they globally affected stock and real estate markets. Globally, firms faced severe losses due to investors’ decreasing confidence, the lack of disposable income, and the drop in consumer spending (Brunnermeier 2009).

The COVID-19 pandemic, one of the worst pandemics since the start of the 20th century, was another recent crisis event. Like the GFC, it resulted in an economic downturn across the globe by reducing economic activities, which had a detrimental impact on firm productivity and profitability (OECD 2020). In the U.S., for example, the gross domestic product fell by 1.3% and 9.5% in the first and second quarters of 2020 (OECD 2020). A global pandemic of this size has a severe impact on firms, their supply chains, and all parts of economic and social life. Consequently, the GFC and the COVID-19 pandemic offer two unique settings for studying organizational resilience (DesJardine et al. 2019; Buyl et al. 2019). Both crises have severely affected firms across all industries, resulting in different degrees of losses and recovery.

### Sample

To test our hypotheses, we collected data on 462 firms in the Standard & Poor’s 500 and their CEOs during the 2008 GFC and the COVID-19 pandemic in 2020. We focused on public firms to analyze stock prices’ movement in response to the two crisis events. In line with prior research, we used September 17, 2008 as the GFC’s starting date, which followed on the heels of Lehman Brothers’ bankruptcy filing on September 15, and the U.S. Federal Reserve’s bailout of AIG at midnight on September 16, 2008 (DesJardine et al. 2019; Sajko et al. 2021). We used February 3, 2020 as the COVID-19 pandemic’s starting date, following the U.S. administration’s declaration of a public health emergency due to the Coronavirus outbreak (AJMC 2021). The announcement came three days after the WHO declared a Global Health Emergency due to 9800 cases of the virus and more than 200 confirmed deaths globally.

We collected our data from several sources. We first gathered transcripts of CEOs’ quarterly conference calls with analysts via the Nexis Uni Database to measure their individual FTD. We did so by developing a sophisticated program that allows us to distinguish between the different parts of the call (presentation and Q&A), and to deal with each speaker (CEO, TMT member, analyst, or operator) separately. We also collected stock data from the CRSP Stock Database and additional financial data from Compustat.

Following established upper echelons research (Michel and Hambrick 1992; McNamara et al. 2002), we identified a TMT as a team of top executives, such as the C and EVP/SVP positions that BoardEx identified by relying on data from DEF14A statements. Thereafter, we identified and removed CEOs when computing the TMT variables. Our identification of TMT members is consistent with other upper echelon studies (Fredrickson et al. 2010; Andrus et al. 2019). We collected additional biographical data on CEOs and TMT members from BoardEx, CapitalIQ, and Bloomberg, as well as also hand-coded missing data from DEF14A statements, the press, or company releases.

Our baseline population covered firms included in the S&P 500 in 2008 and 2020. The main restriction criterion was that to measure a CEO’s FTD, the CEO had to be present during conference calls in the years prior to the crises (respectively, 2007 and 2019). However, CEOs are not always present during these quarterly conference calls. This meant we had a final sample of 290 CEOs for the 2008 GFC and 360 CEOs for the 2020 COVID-19 pandemic.

We followed prior organizational resilience research (Gittell et al. 2006; DesJardine et al. 2019) and measured two distinct dimensions of organizational resilience on the basis of the stock price data: the severity of the loss due to the crises and the time required to recover from them. In part 1, we analyze how a CEO’s FTD affects a firm’s ability to prevent severe economic losses. In part 2, we examine how a CEO’s FTD affects a firm’s ability to recover from both systemic shocks. Since our theoretical suggestions depend on explaining two different mechanisms (i.e. stability vs. flexibility) and require distinct estimation techniques, we outline the two methodological approaches and their results separately.

## Methods

### Empirics Part 1: Addressing Stability

#### Variables

##### Dependent Variable

Following prior research (DesJardine et al. 2019; Sajko et al. 2021), we defined the *severity of loss* as the absolute percentage change in a firm’s stock price between the closing price before the crisis (i.e., on September 16, 2008/February 3, 2020) and the lowest point reached in the 12 months following these dates. The duration was limited to a year to minimize the possibility of other factors affecting the stock prices (DesJardine et al. 2019).

##### Independent Variable

We used quarterly earnings calls, during which a CEO comments on events and discusses the strategy’s implications for the firm’s future performance, to examine a CEO’s *future temporal depth.* While the first part of these calls is a cautious preparation for the announcement, the second part comprises a question and answer section, mirroring a CEO’s unpretentious use of language and thoughts (Lee 2015; Pan et al. 2018).

We utilized ML and secondary data to assess a CEO’s FTD. By building on studies using computer-aided text analysis (Pan et al. 2018), we identified each temporal expression that a CEO used during a conference call and normalized its semantics. We used the following phases to extract and normalize the temporal expressions (Strötgen and Gertz 2010, 2016): (1) the extraction, (2) the normalization, (3) the disambiguation phase, (4) the cleaning phase, and, finally, (5) the specification of ambiguous temporal expressions. Following TimeML (Pustejovsky et al. 2010)—the standard markup language for temporal annotation—we grouped temporal expressions into four types: date, time, duration, and set. We placed the time and date expressions (e.g., “8 p.m.” or “June 27, 2021”) on a timeline. Generically used and vague expressions such as “some weeks later,” were the exceptions. In this regard, we specified the temporal relationship as an anchor time, which was the call’s date. We used expressions of the type duration to inform an interval’s length (e.g., “four months” in “She has been waiting for the new service to be launched for four months”), and expressions of the type set providing information about an event’s periodical aspect (e.g., “once a week” in “He talks to the CEO once a week”). For instance, the temporal expression (i.e. date) in the following quote from Aetna Inc 2012 Q4 held on January 31, 2013, namely “Shawn Guertin will become Aetna’s Chief Financial Officer effective from February 25” translates, for instance, into TimeML format 2013-02-25.

We ensured that the FTD measured precision by undertaking a validation study with an interdisciplinary team of linguists Ph.D. students, computer scientists, and business students, who all coded 8350 temporal expressions in 50 conference calls manually. This approach confirmed our machine learning approach’s reliability as being more than 80%.

To measure a CEO’s FTD, we estimated the temporal distance between the temporal expressions identified in the calls and the anchor date (i.e. the date of the actual call). We used the values of a CEO’s future temporal distances and calculated the average of each call’s temporal distances by dividing the sum of all the temporal distances by the number of all distances that the CEO used. We aggregated these distances from the quarterly calls to a yearly CEO FTD score by using the average of all the quarters in the respective year.

##### Moderator

We measured the *TMT functional diversity* as the Blau index (Blau 1977) of TMT members’ previous work experience in a specific function (Nielsen and Nielsen 2013). We first determined in which of the following categories the executives had spent most of their career by calculating their work periods in Marketing, Sales & Customer Service; Human Resource & Personnel; Information Systems; Planning & Administration; Public Affairs; General Management & Strategy; Academic Research; Operations & Production; Finance & Risk Management; Accounting; Legal & Law; R&D, and Engineering. Thereafter, we calculated the team’s diversity score with the Blau index. We used the function $\mathrm{B}=\left[1-\sum \left(\mathrm{pi}\right)2\right]$, where p is the percentage of members in the i^th^ group (i.e., function). We focused on functional experience diversity since a TMT’s diverse experience is a core mechanism that benefits a CEO by contributing specific functional knowledge and skills (Waller et al. 1995; Kraatz and Moore 2002).

##### Control Variables

We controlled for several CEO characteristics that might influence organizational resilience. Crisis research has suggested that firms led by powerful CEOs perform worse, because the decision making structures at the top of the organization are less dispersed (Gupta et al. 2018). Consequently, we controlled for *CEO duality*, which we captured with a variable coded 1 if the newly appointed CEO also holds the position of chairman, and 0 otherwise (Zajac and Westphal 1996). We measured the *CEO age *as the years since the CEO’s date of birth, and the *CEO functional background* as the function (based on the top executive’s job title) that this executive has fulfilled the longest (Michel and Hambrick 1992). We used the full bibliographical information of the executives’ career history, which is available from BoardEx, and summed the number of years they spent in each functional background (e.g., finance, marketing). We used the functional backgrounds in which they spent the most working years as their dominant functional background, since this impacts their performance during a crisis (Mio et al. 2016). We also controlled for the *CEO tenure* by counting the number of years a CEO fulfilled this position (Henderson et al. 2006).

In line with prior upper echelons research, we also included several TMT controls. We measured the *TMT size* as the total number of TMT members in a relevant year (Siegel and Hambrick 2005). We control for the *TMT age diversity*, and *TMT tenure diversity* by measuring them as, respectively, the coefficient of the variation of the continuous variables of each TMT member’s age and tenure (Nielsen and Nielsen 2013). We measured the *TMT industry diversity* as the Blau index of TMT members’ previous dominant, industry-specific (4-digit SIC) experience throughout their career history (Nielsen and Nielsen 2013). We used additional TMT controls for our robustness checks.

We controlled for several variables that might have an impact on organizational resilience: *Firm size* was measured as the number of employees (in thousands) (Tushman and Rosenkopf 1996). We also controlled for the *firm age *(Haleblian and Finkelstein 1993), i.e. the number of years that had elapsed since the firm’s founding. Both variables were then logarithmically transformed. The older the firm, the greater the likelihood that it had acquired resources (e.g., human capital) to manage negative events. *Capital intensity* refers to a firm’s total capital expenditures deflated by its total assets. According to Gittell et al. (2006), more capital-intensive firms perform worse than those that are less capital intensive. To control for the differences in the financial criteria, we also included *profitability*, which is the ratio of the operating income before depreciation and amortization (earnings before interest, taxes, depreciation, and amortization) to the book value of total assets. More profitable firms are more likely to receive investor support in times of crisis (Duchin et al. 2010). We followed extant research (Souder and Bromiley 2012; Martin et al. 2016; Bromiley 1991) and defined the *available slack* resources as a measure of the resources that the firm holds, which could act as a buffer against economic turbulence, provide funds for adaptation, or could finance strategic investments. Consequently, this measure captures a firm’s liquid assets and is measured as a firm’s cash and short-term securities (Souder and Bromiley 2012). We also controlled for* firm performance, *measured as the industry-adjusted ROA. We further controlled for *organizational crisis experience* as a dichotomous variable assessing whether a firm has dealt with a performance crisis in the previous three years. We created dummy variables to control for the *industry*, using the 4‑digit Standard Industry Classification (SIC) code. The cyclicality of industry classes differs, since investors tend to turn to defensive stocks (such as utilities) in adverse economic environments. We also included a dummy variable indicating the specific crisis with a variable coded 1 for the GFC, and 0 for the COVID-19 pandemic.

#### Analysis

We used ordinary least squares (OLS) regression with Newey-West estimator (Newey and West 1987) to account for heteroscedasticity and autocorrelation. The mean of the variance inflation factors (VIF) across all the models is 1.79. This number is well below the threshold level (Neter et al. 1996), which suggests that multicollinearity is not a problem.

#### Results

Table 1 reports the descriptive statistics and pairwise correlations, while Table 2 reports our OLS models’ results. Model 1 is the baseline model of the severity of loss, comprised of the control variables. Consistent with prior research (e.g., DesJardine et al. 2019; Sajko et al. 2021) the TMT age diversity (*β* = 0.356, *p* = 0.011), the firm size (*β* = 0.019, *p* = 0.001) and the crisis dummy (*β* = 0.138, *p* = 0.000) have significant positive coefficients, while the CEO tenure (*β* = −0.003, *p* = 0.003), CEO functional background (*β* = −0.008, *p* = 0.021), the TMT size (*β* = −0.012, *p* = 0.000), the profitability (*β* = −0.306, *p* = 0.010), and the firm performance (*β* = −0.376, *p* = 0.000) have significant negative coefficients.

**Table 1:** Descriptive Statistics and Correlation Matrix

Variables	(1)	(2)	(3)	(4)	(5)	(6)	(7)	(8)	(9)	(10)	(11)	(12)	(13)	(14)	(15)	(16)	(17)	(18)
(1) Severity of Loss	1.00	–	–	–	–	–	–	–	–	–	–	–	–	–	–	–	–	–
(2) CEO FTD	−0.06	1.00	–	–	–	–	–	–	–	–	–	–	–	–	–	–	–	–
(3) CEO Tenure	−0.09	0.00	1.00	–	–	–	–	–	–	–	–	–	–	–	–	–	–	–
(4) CEO Age	−0.05	−0.02	0.41	1.00	–	–	–	–	–	–	–	–	–	–	–	–	–	–
(5) CEO Func. Backgr	−0.08	0.06	−0.13	−0.03	1.00	–	–	–	–	–	–	–	–	–	–	–	–	–
(6) CEO Duality	0.00	0.04	0.34	0.23	−0.10	1.00	–	–	–	–	–	–	–	–	–	–	–	–
(7) TMT Size	−0.01	−0.02	−0.10	−0.09	−0.01	0.09	1.00	–	–	–	–	–	–	–	–	–	–	–
(8) TMT Tenure Div	0.01	0.00	−0.14	−0.10	0.02	−0.08	0.27	1.00	–	–	–	–	–	–	–	–	–	–
(9) TMT Func. Div	−0.10	−0.01	0.03	−0.02	0.03	−0.03	0.19	0.05	1.00	–	–	–	–	–	–	–	–	–
(10) TMT Ind. Div	0.02	0.04	−0.15	−0.03	−0.03	−0.07	0.15	0.26	−0.05	1.00	–	–	–	–	–	–	–	–
(11) TMT Age Div	0.14	−0.03	0.00	−0.08	−0.01	−0.02	0.18	0.11	−0.01	0.06	1.00	–	–	–	–	–	–	–
(12) Capital Int	0.06	0.10	0.02	0.02	−0.06	0.05	0.03	0.05	0.11	−0.10	0.02	1.00	–	–	–	–	–	–
(13) Profitability	−0.20	−0.05	0.00	−0.06	−0.06	0.02	0.07	0.03	0.14	−0.22	0.00	0.32	1.00	–	–	–	–	–
(14) Available Slack	0.01	−0.08	0.08	−0.06	−0.08	−0.05	0.05	−0.05	0.10	−0.10	0.11	−0.06	0.11	1.00	–	–	–	–
(15) Org. Crisis Exp	0.00	−0.08	−0.05	0.00	−0.01	0.04	−0.04	0.07	−0.06	0.03	−0.04	−0.02	−0.13	−0.09	1.00	–	–	–
(16) Performance	−0.32	0.03	0.02	−0.01	−0.06	0.05	0.06	−0.02	0.10	−0.08	−0.04	0.01	0.51	0.13	−0.13	1.00	–	–
(17) Firm Size (log)	−0.04	−0.04	−0.11	0.12	0.03	0.07	0.28	0.07	−0.04	0.08	−0.15	−0.02	0.06	−0.29	0.02	−0.01	1.00	–
(18) Firm Age (log)	0.01	0.01	−0.13	0.14	0.05	0.14	−0.02	−0.03	−0.15	−0.01	−0.13	−0.14	−0.21	−0.23	0.01	−0.02	0.27	1.00
Mean	0.45	0.34	6.04	56.82	7.57	0.49	6.87	0.55	0.63	0.27	0.12	0.04	0.13	1.76	0.45	0.07	2.84	68.02
S.D.	0.18	0.27	5.41	6.65	1.70	0.50	2.69	0.23	0.16	0.25	0.05	0.05	0.07	1.20	0.50	0.11	1.33	49.15

Correlations with magnitude larger than 0.07 are significant at the *p* < 0.05 level

**Table 2:** OLS Estimates: A CEO’s FTD and Severity of Loss

	(1)	(2)	(3)
VARIABLES	Severity of Loss	Severity of Loss	Severity of Loss
CEO Tenure	−0.003**	−0.003**	−0.003**
	(0.001)	(0.001)	(0.001)
CEO Age	0.001	0.001	0.001
	(0.001)	(0.001)	(0.001)
CEO Functional Background	−0.008**	−0.007**	−0.007**
	(0.003)	(0.003)	(0.003)
CEO Duality	−0.013	−0.011	−0.012
	(0.014)	(0.014)	(0.014)
TMT Size	−0.012***	−0.012***	−0.012***
	(0.003)	(0.003)	(0.003)
TMT Tenure Diversity	0.008	0.008	0.008
	(0.028)	(0.027)	(0.027)
TMT Industry Diversity	−0.004	−0.002	−0.002
	(0.027)	(0.027)	(0.027)
TMT Age Diversity	0.359**	0.350**	0.362**
	(0.141)	(0.142)	(0.141)
Capital Intensity	0.137	0.168	0.173
	(0.142)	(0.146)	(0.144)
Profitability	−0.306***	−0.349***	−0.361***
	(0.118)	(0.121)	(0.120)
Available Slack	0.005	0.004	0.005
	(0.006)	(0.006)	(0.006)
Organizational Crisis Experience	−0.014	−0.018	−0.016
	(0.012)	(0.012)	(0.012)
Performance	−0.376***	−0.367***	−0.358***
	(0.061)	(0.061)	(0.061)
Firm Size (log)	0.019***	0.018***	0.019***
	(0.006)	(0.006)	(0.006)
Firm Age (log)	−0.000	−0.000	−0.000
	(0.000)	(0.000)	(0.000)
Crisis Control	0.138***	0.137***	0.137***
	(0.016)	(0.016)	(0.015)
Industry Control	−0.000***	−0.000***	−0.000***
	(0.000)	(0.000)	(0.000)
TMT Functional Diversity	0.056	0.053	0.205***
	(0.048)	(0.048)	(0.074)
CEO Future Temporal Depth	–	−0.0518**	0.210**
	–	(0.025)	(0.104)
CEO FTD × TMT Functional Diversity	–	–	−0.397**
	–	–	(0.159)
Constant	0.455***	0.485***	0.391***
	(0.084)	(0.087)	(0.094)
*N*	650	650	650

Standard errors in parentheses*** *p* < 0.01, ** *p* < 0.05, * *p* < 0.1

Hypothesis 1 predicted that a CEO’s longer FTD is negatively associated with the severity of loss. Model 2 in Table 2 indicates a negative and significant coefficient (β = −0.051, *p* = 0.047). Hypothesis 1 is therefore supported. Hypothesis 3 predicted that a TMT’s functional experience diversity is associated with an increase in the negative effect of CEOs’ FTD on the severity of loss. Model 3 suggests that the hypothetical parameter estimate for CEO FTD (*β* = 0.210, *p* = 0.044) and the interaction of CEO FTD and TMT functional diversity (*β* = −0.397, *p* = 0.013) are both statistically significant.

**Fig. 1:**
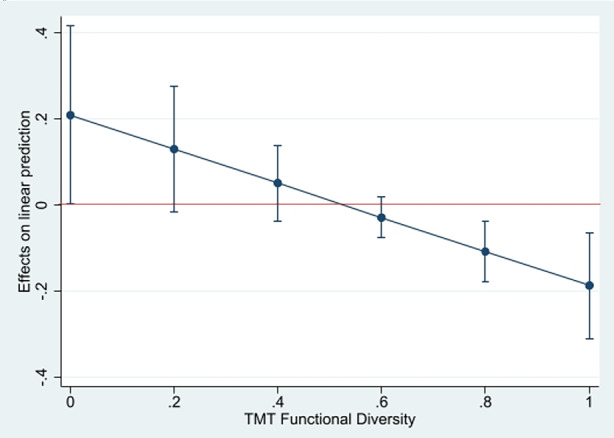
Marginal Effects of CEO FTD with 95% Confidence Intervals. The *horizontal line* represents the value 0. The *vertical lines* represent the 95% confidence interval for any given marginal effect

We followed recent research (Busenbark et al. 2022a) by applying the marginal effects approach to further assess the moderating effect. Table 3 displays the marginal effects of CEO FTD on the severity of loss deviation (i.e.,dy/dx) at different values of TMT diversity, ranging from very low diversity (0, i.e. a homogenous team) to very high diversity (1, i.e. a very diverse team). As Fig. 1 and Table 3 illustrate, the hypothesized relationship only becomes statistically significantly different from 0 at values for TMT diversity lower than 0.25 (dy/dx = 0.111, *p* = 0.093) and higher than 0.65 (dy/dx = −0.048, *p* = 0.037). Hypothesis 1 is thus only partially supported, especially since Model 3 shows a positive coefficient for the relationship between CEO FTD and the severity of loss. The marginal effects in Fig. 1 are consistent with the coefficient of the interaction insofar as the relationship between CEO FTD and severity of loss decreases as TMT diversity increases from values of 0.65 and above, indicating partial support for Hypothesis 3.

**Table 3:** Marginal effects corresponding to the OLS model

TMT Functional Diversity	dy/dx	*p*-value
0 (low diversity)	0.210	0.044
0.15	0.150	0.063
0.25	0.111	0.093
0.35	0.071	0.167
0.5	0.011	0.723
0.65	−0.048	0.037
0.75	−0.088	0.003
0.85	−0.128	0.002
1(high diversity)	−0.187	0.003

### Empirics Part 2: Addressing Flexibility

#### Variables

##### Dependent Variable

We calculate the *time to recovery* as the time it took a firm to fully recover after a crisis event, i.e. until the firm’s monthly stock price reached its pre-crisis level (i.e., since the closing price on September 16, 2008/February 3, 2020) (DesJardine et al. 2019).

##### Independent Variable

A CEO’s FTD was operationalized as discussed above.

##### Moderation

We operationalized *organizational crisis experience* as a dichotomous variable to assess whether a firm had encountered a performance crisis during the previous three years (Tushman and Rosenkopf 1996). We measured performance crises by examining the change in a firm’s industry-adjusted ROA. If the ROA had decreased over three successive quarters, the variable took the value 1. We applied a time window of three years before the crisis events, since learning to build and develop resilience is not a static or linear process (Haunschild et al. 2015). The ability to learn from crises weakens over time (Williams et al. 2017).

##### Control Variables

We included the same set of control variables as in Part 1. In addition, because some of our sample firms had experienced a CEO change before the recovery, we excluded those firms from the sample to guarantee that we followed an identical CEO population across all the analyses. We reran this analysis by including firms with CEO changes during their recovery. This means that the resulting sample might be biased toward certain types of CEOs who are less likely to leave during the recovery, which could also affect organizational resilience. We used the two-stage Heckman selection model (1979) to control for this potential bias. Following extant research (Keil et al. 2022), we pooled our observations of the firm-year level, including those without CEO appointments, to predict the probability of a firm changing its CEO. In line with research on CEO succession (Zhang and Qu 2016), we included pre-succession firm performance, firm size and age (log-transformed), predecessor CEO founder’s status, the predecessor CEO’s tenure, and age (log-transformed), the industry’s CEO succession rate, and year fixed effects as predictors. We used a probit model with clustered standard errors at the firm level to calculate the probability of CEO succession. We used the predicted scores to calculate the inverse Mills ratio, which we then used in the regressions to control for sample selection.

#### Analysis

We used a Cox regression model with time-varying covariates (Sajko et al. 2021). This has the advantage of not making assumptions about the distribution of the survival function. This specification makes a model more flexible and robust when it is hard to specify a specific distribution of the times to recovery. In our context, survival analysis estimated the hazard rates by regressing the recovery times on the lagged values of our explanatory variable (a CEO’s FTD). Survival analysis was suitable for our data, since it accommodates right censoring and skewness of the survival data (DesJardine et al. 2019).

#### Results

Model 1 in Table 4 is the baseline model of our survival analysis results regarding the time to recovery and comprising the control variables. Consistent with prior research (e.g., DesJardine et al. 2019), TMT functional diversity (β = 1.844, *p* = 0.005), and TMT tenure diversity (β = 0.913, *p* = 0.097) have significant positive coefficients, while the industry (β = −0.000, *p* = 0.014) and crisis dummies (β = −1.072, *p* = 0.000) have a significant negative coefficient.

**Table 4:** Cox Survival: A CEO’s FTD and Time to Recovery (2nd stage)

	(1)	(2)	(3)
VARIABLES	Time to Recovery	Time to Recovery	Time to Recovery
CEO Tenure	0.004	−0.003	0.000
	(0.025)	(0.025)	(0.025)
CEO Age	−0.021	−0.026	−0.027
	(0.022)	(0.022)	(0.021)
CEO Functional Background	0.021	0.044	0.046
	(0.048)	(0.050)	(0.053)
CEO Duality	−0.071	0.032	0.023
	(0.216)	(0.227)	(0.230)
TMT Size	−0.048	−0.043	−0.040
	(0.041)	(0.042)	(0.042)
TMT Tenure Diversity	1.018*	1.184**	1.166**
	(0.567)	(0.574)	(0.579)
TMT Functional Diversity	1.948***	1.752***	1.690**
	(0.665)	(0.664)	(0.661)
TMT Industry Diversity	0.162	0.296	0.271
	(0.389)	(0.392)	(0.390)
TMT Age Diversity	−1.181	−2.060	−1.773
	(1.995)	(2.014)	(2.022)
Capital Intensity	−3.216*	−2.066	−2.108
	(1.930)	(1.905)	(1.895)
Profitability	0.136	−1.074	−0.922
	(1.736)	(1.806)	(1.824)
Available Slack	−0.077	−0.079	−0.083
	(0.070)	(0.069)	(0.071)
Performance	0.687	1.042	0.973
	(0.833)	(0.899)	(0.881)
Firm Size (log)	0.097	0.100	0.094
	(0.072)	(0.069)	(0.069)
Firm Age (log)	−0.002	−0.002	−0.002
	(0.002)	(0.002)	(0.002)
Environmental Munificence	1.624	1.304	1.541
	(1.930)	(1.858)	(1.850)
Industry Control	−0.000**	−0.000**	−0.000**
	(0.000)	(0.000)	(0.000)
Year Control	0.098***	0.104***	0.107***
	(0.020)	(0.020)	(0.020)
End. Control CEO Change	−0.379	−0.570	−0.592
	(0.434)	(0.431)	(0.422)
Org. Crisis Experience	−0.017	−0.132	−0.612**
	(0.161)	(0.167)	(0.284)
CEO Future Temporal Depth	–	−0.736***	−0.985***
	–	(0.282)	(0.294)
CEO FTD × Org. Crisis Exp	–	–	1.367**
	–	–	(0.568)
Number of firms	567	567	567
Number of firms failed	169	169	169
Log-Likelihood	−684.2	−682.0	−680.5
Wald Chi-squared	64.11	70.12	78.48

Standard errors in parentheses*** *p* < 0.01, ** *p* < 0.05, * *p* < 0.1

Hypothesis 2 predicted that a CEO’s longer FTD increases the time to recovery. Model 2 indicates that a firm with a CEO with a longer FTD is more likely to recover slower from adversity than those with a CEO with a shorter FTD (*β* = −0.749, *p* = 0.009). A negative coefficient on a covariate in the survival model indicates a lower probability of a faster recovery, indicating that the covariate contributes to lower firm flexibility. Our results indicate that a one-SD increase in a CEO’s FTD results in an 18.74% decrease [exp(coefficient)—1 × 100%] in the probability of recovery, thereby supporting Hypothesis 2. The included endogeneity control has a negative, but insignificant, coefficient (*β* = −0.518, *p* = 0.215), indicating that sample selection bias due to the exclusion of CEO change cases is unlikely.

**Fig. 2:**
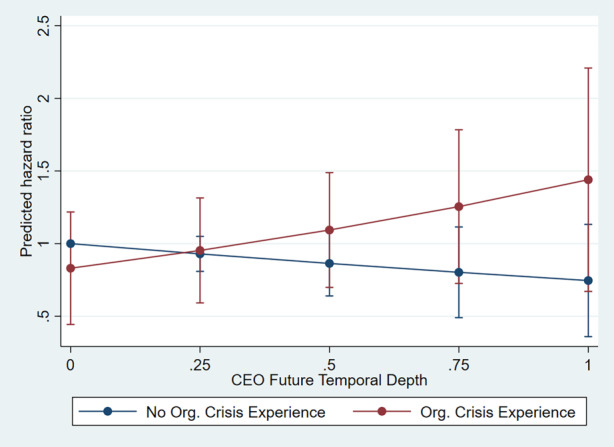
Adjusted Predications of Organizational Crisis Experience with 95% Confidence Intervals

Hypothesis 4 predicted that prior organizational crisis experience mitigates the effect of a CEO’s longer FTD on the time to recover from adversity. Model 3 supports our hypothesis, showing that organizational crisis experience mitigates the effect of a CEO’s longer FTD on the time required to recover (β = 1.334, *p* = 0.018). A positive coefficient on a covariate in the survival model indicates a higher probability of a faster recovery, indicating a contribution of the covariate to higher firm flexibility. Fig. 2 illustrates this relationship.

### Robustness Tests

We conducted a series of robustness tests to verify the main results. We first tested alternative ways to measure a CEO’s FTD. Given the nature of time, we took temporal data of different granularity into account. A CEO, for instance, mentions days, months, or years. To estimate the temporal distance, we needed to ensure that each temporal expression had the same level of granularity and used a date to specify each of them. As a temporal expression in text, we, for example, relate “year” to different dates within a year: the beginning of the year (1/1/2022), the middle of the year (30/06/2022), and the end of the year (31/12/2022). We used different operationalizations in additional analyses, leading to consistent results.

Second, we added several additional controls, none of which changed our results significantly. We controlled for analysts’ FTD by means of the temporal expression they used in the Q&A section, which might influence the temporality of the CEO’s answers. We also controlled for CEO-level factors, such as CEO outsiderness, since research has shown that firms led by insider CEOs outperform those led by outsider CEOs in terms of their ROA during the 2020 COVID-19 crisis period (Haque et al. 2022). Prior research also showed that firms with female CEOs and board chairs were less likely to fail during the GFC (Palvia et al. 2015). We therefore controlled for CEO gender, measured as a dummy variable and coded 1 if the CEO was male. In addition, we controlled for several TMT-level factors, such as a TMT change, since it can harm a TMT’s decision making (Li and van Knippenberg 2021), which could be crucial in times of crisis. We also included the mean of the TMT age, tenure, and educational level, as well as TMTs’ diversity measures, all of which did not change our results significantly.

Third, we applied different time lags to our independent variable. When applying these to our CEOs’ FTD measures, we found that only a CEO’s FTD in the year preceding the crisis event impacted the severity of loss and the time to recovery.

Fourth, we addressed the risk of omitted variables by applying the impact threshold of a confounding variable (ICTV) (Busenbark et al. 2022b), which can be applied as a sensitivity analysis to determine the correlation values at which an estimate of the independent variable becomes sufficiently high enough to be considered “not zero.” In our case, an ITCV value of 0.121 means an omitted variable would need to have an average partial correlation with the independent and dependent variables at a minimum of 0.121 in order to invalidate the causal inference. Higher ITCV scores indicate that an omitted variable would have to have a higher correlation, which suggests it is less likely to suffer from bias inferences (Busenbark et al. 2022b). This means that our findings are less likely to suffer from bias inferences.

Fifth, we addressed different concerns regarding selection bias. We followed recent research in assessing the likelihood of a CEO being present during a conference call (Malhotra and Harrison in press). Given our sample includes only CEOs who participated in earnings calls during our sample period, a selection bias may exist (Certo et al. 2016). We address this by using Heckman’s (1979) two-stage approach. The dataset for this analysis included 11,212 firm-year observations of S&P 500 firms from 2004 to 2020 with financial data available in Compustat. We created a dummy variable equal to 1 for firm-years in which the CEO participated in earnings calls, and 0 otherwise. Following Malhotra and Harrison (in press), we estimated a probit regression in the first stage using CEO, firm, and industry factors that may be related to selection into our sample and then used estimates from this model to calculate the inverse mills ratio. Our findings did not change significantly in the second stage models.

Consistent with previous research on CEO-level traits and organizational resilience (Buyl et al. 2019), we recognize that the decision to hire a CEO with a longer FTD may correlate with the decision to build organizational resilience. It is therefore possible that there were specific variables at the CEO and company level that led to systematic differences in both the choice to select a CEO with a longer FTD and the resulting organizational resilience. Addressing this, we partial out the part of our CEO FTD measure that is the result of such variables (eliminating unobserved heterogeneity). Building on previous resilience research (e.g., Buyl et al. 2019), we regress our FTD measure against carefully chosen CEO- and firm-level variables related to the conditions that might have affected the selection of CEOs with a longer FTD in the first place and that, in turn, might be responsible for the resilience afterward. We used the estimates from this model to calculate the inverse mills ratio, which we included in the second stage. The results were similar to our main results shown in Tables 2 and 4.

Sixth, we treated each crisis separately and re-ran our models for the GFC and the COVID-19 pandemic independently. Our main findings for H1 held during both crisis events. However, the interaction effect of TMT functional diversity hypothesized in H3 only yielded significant results for the GFC, but not the COVID-19 pandemic (*β* = −0.294, *p* = 0.193). To compare both crises, we combine the findings of both crises in one model in our main results, while accounting for autocorrelation and heteroscedasticity.

Seventh, we tested the key assumptions of the Cox proportional hazard models that each predictor variable’s effect remains constant (i.e., it is proportional) over time. This was intended to be more certain that the effect of a CEO’s FTD on a firm’s recovery time is not a function of time if the proportionality assumption holds. We tested for Schoenfeld residuals by applying the Stata “estat phtest” command. The test failed to reject the null hypothesis, which suggests that it is unlikely that time-dependent effects affected our results (*p* > 0.05).

Eighth, in line with prior research (Sajko et al. 2021) we estimated Cox proportional hazard models with shared frailty. Shared frailty models are survival data analog to random effects models. Frailty is a latent random effect used to model within-group correlation, thereby accounting for unobserved group heterogeneity. Our findings remained largely consistent after the specification of the shared frailty.

Ninth, we reran the analyses, including those firms with a CEO change during the recovery period. We replaced the sample selection control with a dummy variable (“CEO change”) that equals 1 if the firm experienced a CEO change during our examined window of time. We found that a CEO’s longer FTD has a negative effect on a firm’s recovery time (*β* = −0.654, *p* *=* 0.008) with the CEO change variable yielding no significant effect, thereby providing additional support for Hypothesis 2. Moreover, our main finding regarding Hypothesis 4 that organizational crisis experience mitigates the relationship between CEO’s FTD and the time to recovery (*β* = 0.836, *p* *=* 0.099) was supported.

Tenth, prior research stressed the importance of available company resources in anticipating potential adversity (Carmeli and Markman 2011; George 2005). These flexible resources are essential to enable a CEO to take timely corrective actions and maintain flexibility (Rahmandad and Repenning 2016). We tested this assumption by studying the effect of available financial slack on the relationship between a CEO’s longer FTD and the time to recovery from adversity, finding that available slack resources allow a CEO with a longer FTD to recover quicker (*β* = 0.644, *p* *=* 0.055).

Lastly, we repeated our analyses, using different time windows, such as 18 instead of 12 months in respect of both dimensions of resilience, but found no significant differences in our results. Overall, these additional tests support our results’ robustness.

## Discussion

In this paper, we aimed at understanding the role a CEO’s FTD plays in organizational resilience by focusing on the GFC and the COVID-19 pandemic. Our empirical findings show that a longer CEO FTD is associated with less severe economic losses but a slower recovery from an economic shock. On the one hand, such a CEO’s proactive preparation for adversity could help strengthen the organization’s ability to minimize the severity of economic losses. On the other hand, a CEO with a longer FTD favors future benefits over quick results and prompt solutions (Das 1987), which might make such a CEO more cautious about taking immediate action to recover from a crisis fast.

We also found that a TMT’s functional diversity is associated with less severe losses, such that a CEO with longer FTD, who can draw from a functionally diverse TMT, faces less severe losses. We attribute these findings to TMT functional diversity’s role in processing and sharing multiple function-specific experiences and viewpoints, which enables a CEO with a longer FTD to adequately scan the environment and prepare for adversity across different functions. Our results also showed that organizational crisis experience mitigates the effect of a CEO’s FTD on the time to recovery, such that a firm led by a CEO with a longer FTD, who can draw on prior organizational crisis experience, takes less time to recover. Organizational crisis experience seems to foster organizational flexibility by providing a pool of prior organizational responses and timely reactions to adversity, which might push a CEO with a longer FTD to respond more rapidly to a crisis. In the following, we discuss the theoretical implications of these findings for research on organizational resilience and the upper echelons perspective.

### The Role of a CEO’s Temporal Cognition in Organizational Resilience

Our study offers a novel view of a CEO’s role in preparing for and adjusting to adversity. It contributes to organizational resilience research (DesJardine et al. 2019; Ortiz-de-Mandojana and Bansal 2016; Linnenluecke 2017) by uncovering its cognitive underpinnings (Felin et al. 2015). Prior research showed that organizational antecedents, such as flexible financial resources (Gittell et al. 2006), innovation (Reinmoeller and Van Baardwijk 2005), and good governance (Carmeli and Markman 2011) increase organizational resilience. However, scholars have only recently started examining top executives’ role in fostering resilience (Buyl et al. 2019; Sajko et al. 2021). We extend this line of research by introducing the central role that a CEO’s temporal cognition plays in both dimensions of organizational resilience. Individuals select strategies to deal with crises or change, depending on the time frame they adopt (Mumford et al. 2007), which makes the study of a CEO’s FTD relevant for a better understanding of how they deal with major crises. Our findings suggest that a CEO’s FTD has different effects on the severity of loss and on the time to recover from adversity. A focus on both the short and the long term might therefore be beneficial for organizations facing major crises. These situations often require direct and prompt actions (Fietz et al. 2021). One might imagine that a CEO with a shorter FTD, who fosters efficiency and immediate action (Bearden et al. 2006) would be particularly important in terms of dealing with crises under time pressure. Nevertheless, such a CEO does not consider the distant future (Das and Teng 2001), which is equally important for the long-term strategic actions required to prepare for a crisis.

Our study lays the foundations for a research stream on a CEO’s temporal cognition in strategic decisions under time pressure. Although research on temporal cognition dates back to Lewin’s (1942) view of time and has stimulated rich research in psychology, we still lack an understanding of time’s impact on organizational outcomes, such as resilience. Since the world is changing rapidly and unpredictably, an increasing number of CEOs face pressing decisions, which makes our understanding of a CEO’s temporal cognition central to explaining strategic decisions and their implications for organizations in the short and the long term.

Our study finds that a CEO’s FTD impacts the two dimensions of organizational resilience in opposite directions. A CEO’s FTD helps organizational resilience by reducing losses but hurts organizational resilience in terms of reducing time to recovery. Although scholars stressed the distinct mechanisms that enable organizational resilience, research has not yet uncovered resilience’s temporality. Our paper reveals the role of individual time horizons in organizational outcomes and shows that the two dimensions of organizational resilience depend on different time horizons. This has implications for future research on the construct of organizational resilience by challenging the notion of studying organizational resilience as an overarching construct and revealing the importance of developing a more fine-grained understanding of its dimensions. Future research could benefit from further studying how organizations and their decision makers can approach the diverging temporal demands of organizational resilience.

### A Contextual Learning Perspective on Organizational Resilience

We offer a contextual learning perspective on organizational resilience by introducing the TMT functional experience diversity and organizational crisis experience as central contingencies of how a CEO can prepare for and adjust to adversity. Our results show that a TMT’s functional diversity is associated with increasing the effect of a CEO’s FTD on the severity of loss, such that a firm led by a CEO with a longer FTD, who can draw from a functionally diverse TMT, faces less severe losses.

In doing so, we shed light on the complementary CEO-TMT roles (Georgakakis et al. 2019; Simsek et al. 2018). In particular, we discuss the complementarity of a CEO’s FTD and the TMT composition. While a CEO’s FTD focuses on information about a certain time span (Das 1991, 1987), TMT functional diversity creates diverse function-specific knowledge (Buyl et al. 2011a; Van Knippenberg and Schippers 2007). Having this broad spectrum of different views is particularly useful for a CEO with a longer FTD. Functional diversity helps such a CEO prepare for crises across different functions and ensures that initial crisis signals are brought to the TMT’s notice. While the TMT’s functionally diverse knowledge might not be sufficient to overcome a major crisis, it does help stimulate TMT discussions on potential reactions (Duchek et al. 2020), acknowledges diverse viewpoints in advance (Van Knippenberg and Schippers 2007), and fosters cognitive conflict (Bell et al. 2011) in preparation for adversity. Simultaneously, owing to the overarching long-term direction that a CEO with a longer FTD is likely to set for a firm, such a CEO and TMT are likely to find common ground on how to prepare for adversity. Thus, a CEO with a longer FTD is likely to provide the long-term direction, but a diverse TMT determines the scope for open debate, both of which help with decision making regarding preparing an organization for adversity.

We also found that organizational crisis experience mitigates the effect of a CEO’s FTD on the time to recover, such that a firm led by a CEO with a longer FTD, who can draw on prior organizational crisis experience, recovers faster. Extant resilience research has suggested that organizations can develop resilience by learning from experience with adversity (Williams et al. 2017; Weick et al. 2008). These scholars argue individual organizational members create learning by encoding new information, adjusting their mental models, and encoding new knowledge into organizational routines (Madsen 2009). Our findings imply that a better understanding of organizational weaknesses and crisis causes, which increases with prior crisis experience, allows organizations to respond to them more effectively (Kovoor-Misra and Nathan 2000). The experience with prior organizational crises helps a CEO with a longer FTD to apply adequate and timely responses to adversity—despite a focus on the long term.

We also contribute to upper echelons research (Hambrick 2007) by studying the important role that a CEO’s FTD plays regarding resilience in times of crisis. The growing research on a CEO’s temporal cognition examines its role in different strategic decisions (Das 1987; Nadkarni et al. 2016) and focuses on planned decisions. We, conversely, focus on the role of a CEO’s FTD in situations requiring quick decisions under time pressure. In such situations, a CEO with a longer FTD has both a distinct and a differential influence on the two types of resilience: it fosters a firm’s preparation for crises but limits its ability to respond quickly to a crisis. Our study shows the importance of a CEO’s FTD when making strategic decisions under time pressure and the complementary role that a functionally diverse TMT can play by influencing organizational resilience.

Lastly, we assess a CEO’s FTD by applying state-of-the-art ML techniques (Menon et al. 2018). We show ML’s potential when studying executive cognition (Menon et al. 2018) by introducing a novel measure of a CEO’s FTD. In line with the latter, research on language and time in strategic management (Crilly 2017) suggested that applying a cognitive-linguistic lens lends itself to studying how managers construe numerous abstract concepts that are core to strategic thinking. We enrich this research by showing that a CEO’s temporal cognition is conveyed by language and eventually translates into profound organizational implications—in our case organizational resilience. This insight should pave the way toward a more automated measurement of a CEO’s cognition in secondary data.

### Managerial Implications

Our findings have important practical implications for boards of directors’ governance of top executives and their strategic actions during crises. A CEO’s longer FTD does not only have strategic planning benefits (Das 1991), but it can also have benefits for building an organization’s ability to withstand external adversity. However, this FTD diminishes an organization’s ability to react fast and flexibly. This implies that a board needs to closely monitor a CEO with a specific FTD, particularly in times of crisis. Since TMT diversity decreases the severity of loss, it is important for those who determine the design of a firm’s TMT—whether this is the CEO or the board of directors or both—to be aware of TMT diversity’s influence, particularly if their CEO has a longer FTD and there is a need to prepare for a potential crisis. Moreover, our findings have implications for CEO selection. If a board anticipates turbulence or is currently looking for a CEO to master a crisis, it needs to govern and continuously evaluate the CEO’s cooperation with the TMT to leverage its expertise.

### Limitations and Future Research

Like most studies, our research also has its limitations. First, resilience scholars suggest that resilience is path-dependent and might improve over time. We addressed this by taking the firm’s age into account. Nevertheless, future research could develop a more fine-grained measure of how past shocks affect an organization by, for instance, differentiating between external and internal shocks.

Second, while our focus is on the antecedents of organizational resilience, research maintains that an organization can only be as resilient as its teams and employees (Hartmann et al. 2021; Carmeli et al. 2012; Gucciardi et al. 2018). It would therefore be interesting to study CEOs’ resilience by focusing specifically on how they become resilient and how this process influences the way they address organizational resilience.

Third, we operationalized organizational resilience as stock prices’ reactions to external shocks. Although stock prices are the best measures with which to ascertain resilience in times of adversity (Hillmann and Guenther 2021; DesJardine et al. 2019), financial markets are very sensitive to firm-specific factors, which could confound potential results. Future research could therefore explore new pathways toward operationalizing organizational resilience, such as the differences in organizations’ resource allocations. Along this line, future research could also develop appropriate measures to study resilience and changes in resilience over longer periods.

Fourth, we need to better understand how a CEO’s FTD interacts with other personality traits, such as extroversion or narcissism in terms of their influence on organizational resilience over time. This is particularly interesting, because prior research has shown that CEO greed and narcissism impact organizational resilience (Sajko et al. 2021; Buyl et al. 2019). In line with this, we acknowledge that a CEO’s FTD is a relatively stable construct and the FTD only in the year prior to the crisis had a significant effect on the dependent variables.

Fifth, since the COVID-19 pandemic is a recent crisis event that started in 2020, we could only collect data for a time frame of 18 months after the onset of the crisis. Therefore, our analysis of the time to recover might suffer from a censoring problem with respect to the COVID-19 pandemic. Future research could extend the time frame and compare how organizations recover after different types of systemic crises.

Lastly, using a TMT’s functional experience diversity, we focus on the use of TMT demographics. Owing to constraints regarding data availability, we could not study internal process-oriented TMT variables (Lawrence 1997). Despite this limitation, we believe that TMT members’ functional background—often regarded as a more proximal indicator of skills (e.g., Bunderson and Sutcliffe 2002)—is more relevant than distal indicators, such as age or nationality. Future research could use proxies directly targeted at CEO-TMT interactions and dynamics. For instance, how does a CEO with a longer FTD leverage a TMT’s human capital?

## Conclusion

Our paper is the first to introduce a novel framework of the role a CEO’s FTD plays in organizational resilience. We integrated research on organizational resilience (Hillmann and Guenther 2021; Conz and Magnani 2020) and upper echelons (Hambrick 2007; Buyl et al. 2019) to examine the effect of CEO’s FTD on organizational resilience, thereby unveiling its cognitive underpinnings. By applying a novel ML approach to measure a CEO’s FTD by means of managers’ conference calls with analysts, we tested our hypotheses with a sample of 462 S&P500 firms during two recent crisis events: the 2008 GFC and the COVID-19 pandemic. Our findings confirmed that a CEO’s longer FTD is associated with less severe economic losses, but longer time to recovery. However, a firm is associated with even less severe losses if a CEO with a longer FTD has a functionally diverse TMT at hand and recovers faster if it has recently experienced an organizational crisis.

Our study offers a more fine-grained understanding of resilience and how a CEO prepares and adjusts the organization in the face of adversity. It contributes to organizational resilience research (Ortiz-de-Mandojana and Bansal 2016; DesJardine et al. 2019) by uncovering its cognitive underpinnings, thereby unveiling the important role that a CEO’s cognition plays in influencing an organization’s resilience. We offer a contextual learning perspective on organizational resilience by emphasizing TMT experience diversity’s and organizational crisis experience’s different effects. We also contribute to upper echelons research (Nadkarni et al. 2016; Chen and Nadkarni 2017) by emphasizing the important role that a CEO’s FTD plays in organizational resilience and a diverse TMT’s importance when collaborating with a CEO to steer a firm through crises. Lastly, we apply state-of-the-art ML techniques (Menon et al. 2018) to develop a novel measure of a CEO’s FTD, which future research on a CEO’s temporal cognition could use.

We hope that our paper provides a foundation for future research on the role a CEO’s temporal cognition plays in preparing for and adjusting to adversity.
